# The Individual Variations in Sperm Quality of High-Fertility Boars Impact the Offspring Production and Early Physiological Functions

**DOI:** 10.3390/vetsci12060582

**Published:** 2025-06-13

**Authors:** Santa María Toledo-Guardiola, Chiara Luongo, Felipe Martínez-Pastor, Cristina Soriano-Úbeda, Carmen Matás

**Affiliations:** 1 Department of Physiology, Faculty of Veterinary Medicine, University of Murcia, Campus Espinardo, 30100 Murcia, Spain; smaria.toledo@um.es (S.M.T.-G.); chiara.luongo@um.es (C.L.); 2 Cell Biology, Department of Molecular Biology, University of León, Campus Vegazana, 24071 León, Spain; f.martinez@unileon.es; 3 Institute of Animal Health and Livestock Development (INDEGSAL), University of León, Campus Vegazana, 24071 León, Spain; 4 Animal Reproduction and Obstetrics, Department of Veterinary Medicine, Surgery, and Anatomy, University of León, Campus Vegazana, 24071 León, Spain; 5 Pascual Parrilla Murcian Biosanitary Research Institute (IMIB—Arrixaca), 30120 Murcia, Spain

**Keywords:** artificial insemination, high-fertility boars, piglets, production, spermatozoa, neonatal health, glucose regulation

## Abstract

Artificial insemination (AI) is essential in intensive pig production, which significantly depends on semen quality from boars selected for health, genetics, and fertility. While AI aims to improve productivity, larger litters often result in smaller and less resistant piglets. Beyond fertility and genetic traits, boars also influence offspring health. This study investigated the relationship between sperm parameters of highly fertile boars and both reproductive outcomes and piglet physiological indicators. Multivariate analysis revealed significant paternal effects on blood markers reflecting organ function, including those of the pancreas, liver, and kidneys, as well as on glucose homeostasis, lipid metabolism, oxidative stress, protein and carbohydrate metabolism, muscle contraction, and neural signaling. Notably, sperm velocity was correlated with mitochondrial function, which is crucial for sperm motility, capacitation, DNA integrity, and embryo development—factors likely linked to healthier, more resilient offspring. Boars transmitting superior sperm velocity, erythropoiesis efficiency, and oxygen transport capacities produced piglets with better glucose regulation, growth, and resistance to neonatal hypoglycemia. These findings underscore the broader impact of sperm quality on offspring vitality and suggest that advanced sperm analysis could improve boar selection and enable more effective, health-oriented breeding strategies.

## Simple Summary

Artificial insemination (AI) is widely used in pig farming to improve productivity, relying on semen from selected healthy and highly fertile boars. However, while AI enables higher productivity, producing larger litters is often accompanied by concomitant challenges, such as more competition between piglets for resources and lighter birth weights associated with higher preweaning mortality and poorer post-weaning growth performance. This study explored how the quality of boar sperm is related to fertility and the piglets’ health. We found that some sperm traits—especially those linked to DNA damage—can be strongly linked to important aspects of piglet health, such as indicators of organ function, metabolism, and resistance to stress. For example, piglets from boars with better sperm quality showed improved blood sugar control, stronger growth, and greater resilience to low blood sugar, a common cause of death in newborn piglets. The study highlights that boar sperm carries more than just genetic traits—it is also linked to the piglets’ growth and survival. Using advanced statistical tools to analyze the relation between multiple variables could help farmers choose boars that produce healthier offspring, leading to more efficient and sustainable pig production.

## 1. Introduction

Artificial insemination (AI) is widely used in intensive pig production systems [1], with farms either purchasing semen doses or producing their own by housing boars. Selecting the right boar involves evaluating genetic quality, health, reproductive performance, and production records. High-quality semen with good sperm characteristics and motility is essential for [2] farm productivity and profitability [2]. Boar performance is confirmed through successive AI and litters, making predictive markers valuable for saving time and costs. Farm’s fertility improvement is crucial in commercial pig production. The number of piglets weaned per sow per year, often a productivity benchmark, has risen from 20 to 30 in recent decades, potentially increasing to 30–40 piglets (reviewed by [3]). However, higher piglet numbers can reduce birth weights, limit colostrum intake, and increase pre-weaning mortality [3]. Thus, higher production does not always translate to higher profitability or better animal welfare.

It has been suggested that the effects of a male’s ejaculate extend beyond fertility, inducing lasting changes in the female immune response and reproductive physiology. Among other things, signals triggered by the ejaculate influence ovulation [4,5] and regulate fetal and placental development to influence offspring viability and phenotype. This provides an epigenetic mechanism by which paternal factors can maximize offspring viability and confer fitness, affecting female reproductive resources’ immediate and future investment. The effects of the spermatozoa and seminal plasma on the female environment thus have far-reaching consequences for offspring, independent of the genetic and epigenetic pathways controlled by sperm (reviewed by [6]). Beyond conventional semen parameters, specific aspects such as sperm DNA fragmentation have been linked to reduced embryo viability and long-term effects on offspring health and performance. These subtle defects may go unnoticed in routine evaluations but can significantly impact reproductive outcomes. Determining the individual boar’s role in AI doses is sometimes challenging due to the routine use of heterospermic doses in pig production. Thus, individual boars that show consistent semen quality but have an effect on certain productive and piglets’ parameters related to physiology and their organs’ function can go unnoticed.

Some studies in different mammal species have demonstrated that factors related to semen extend beyond achieving high fertility and prolificacy. For instance, epigenetic mechanisms exist in humans by which paternal factors can maximize viability and transmit specific fitness to offspring, as well as potential maternal influence on reproductive function [4]. Additionally, paternal diet or exercise improve offspring metabolic health via epigenetic modulation of the germline [5]. In mice, litters sired by seminal vesicle-excised males exhibit altered growth trajectories, with elevated central adipose tissue, hypertension, and reduced glucose tolerance, most evident in males [6]. Most of these analyses have focused on improving knowledge about fertility in the human species, and little is known about it in livestock. Remarkably, little is known about the parental factors that influence the long-term risk of disease in offspring after AI with doses from fertility-proven males. Thus, the objective of this study was to analyze the effect of highly fertile boars with excellent spermatozoa quality and prolificacy from AI centers on performance and hematological parameters in their offspring.

## 2. Materials and Methods

### 2.1. Reagents

Unless otherwise indicated, all chemicals were obtained from Sigma-Aldrich, Inc. (Merk KGaA, Darmstadt, Germany) or Thermo Fisher Scientific (Waltham, MA, USA).

### 2.2. Boars and Sows

This study analyzed the ejaculates from fertility-proven boars from an AI center (Sergal Gestió Ramadera, Lleida, Spain). The boars were of Pietrain German Genetics and 2–3 years old. They were housed in individual pens specially designed for boars, with sawdust covering the ground, in compliance with the European Commission Directive for Pig Welfare. They were fed according to their nutritional requirements, and water was available ad libitum.

Crossbred sows (Large-White × Landrace, Danbred genetic) from a commercial farm (Genera S.L., Lorca, Spain) with similar age and body condition ~3 (on a scale from 1 to 5, where 1 was extremely thin and 5 was extremely fat) were selected by parity (from three to five previous births) with 3.7 ± 0.6 (mean ± SD), weaning-to-estrus interval (WEI) of 3.6 ± 0.5 days (mean ± SD), and lactation length between 24 and 28 days. Finally, they were randomly assigned to AI with one of the boars in the study.

After weaning from the previous litter, the sows were housed in individual gestation crates with ad libitum access to water and 4.0 kg feed per day until the AI. From the AI until day 25 of gestation, sows were fed daily with 2.0–3.5 kg of the same gestation feed. From day 25 until the entry to farrowing, all sows received 2.0 kg/day. From the day of farrowing, sows were fed with 1.0 kg/day, with the ration increasing by 1.0 kg/day until reaching a maximum of 8.0 kg/day.

### 2.3. Semen Collection

The ejaculates were obtained with the animal on a dummy and with the gloved-hand method. The samples were collected in a prewarmed recipient covered with gauze tissue to retain the gel fraction. Both the gel fraction and the pre-sperm-rich fraction of the ejaculates were discarded.

### 2.4. AI Doses Preparation

After collection, the ejaculates were evaluated for volume using graduated tubes and sperm concentration using a calibrated sperm analyzer (Androvision^®^ Minitüb, Tiefenbach, Germany) and then diluted in AndroStar^®^ Plus extender (Minitüb, Tiefenbach, Germany) until reaching a final concentration of approximately 30 × 10^6^ spermatozoa/mL. The AI doses were prepared and treated as standard commercial practices. The AI doses were composed of ~1.8 × 10^9^ spermatozoa in 60 mL of commercial extender, stored in specialized plastic bags at 16 °C in refrigeration until use.

### 2.5. Spermatozoa Analysis

#### 2.5.1. Sperm Motility

The motion parameters of the sperm were determined using a computer-assisted sperm analysis (CASA) system (PROISER R+D S.L., Valencia, Spain) previously validated in our laboratory [7]. The system was connected to a negative phase contrast microscope (Leica DMR, Wetzlar, Germany) and a digital camera (Basler Vision, Ahrensburg, Germany). A 4 μL drop of the samples was placed on a pre-warmed at 38.5 °C 20 μm SpermTrack^®^ chamber (STP-21006, PROISER R+D S.L., Valencia, Spain). The acquisition parameters were set at 25 frames per second at a contrast phase microscope of ×10 magnification. Spermatozoa with VAP < 20 μm/s were considered immotile. Progressive motility was defined as STR > 45%. A minimum of 3 fields per sample were evaluated, with a minimum of 200 spermatozoa counted per field. The motility parameters obtained per sample were total motility (%), progressive motility (%), curvilinear velocity (VCL, µm/s), straight-line velocity (VSL, µm/s), average path velocity (VAP, µm/s), amplitude of lateral head displacement (ALH, µm), percentage linearity (LIN, VSL/VCL ratio, %), percentage straightness (STR, VSL/VAP ratio, %), percentage oscillation (WOB, VAP/VCL ratio, %), and beat-cross frequency (BCF, Hz).

#### 2.5.2. Sperm Viability

The samples were incubated with 3.75 µM propidium iodide (PI; P4170, Sigma) in the dark at room temperature for 10 min and then observed under fluorescence microscopy (Leica^®^ DM4000 LED, Wetzlar, Germany; Ex./Em. 488/655 nm) simultaneously to transmitted light brightfield at ×40 magnification. Two hundred spermatozoa per sample were classified as alive (no fluorescence) or dead (red fluorescence). The sperm viability parameter was expressed as the percentage of alive spermatozoa per sample.

#### 2.5.3. Sperm Acrosome Integrity

The samples were incubated with 2 µM lectin PNA from Arachis hypogaea (peanut) FITC-conjugated (PNA-FITC; L7381, Sigma) in the dark at room temperature for 10 min and then observed under fluorescence microscopy (Leica^®^ DM4000 LED, Wetzlar, Germany; Ex./Em. 488/520 nm) simultaneously to transmitted light brightfield at ×40 magnification. Two hundred spermatozoa per sample were classified according to the status of their acrosome as acrosome-intact (no fluorescence) or acrosome-damaged (fluorescent green) spermatozoa. The Acrosome Integrity parameter was expressed as the percentage of acrosome-intact spermatozoa per sample.

#### 2.5.4. Sperm Mitochondrial Activity

The samples were analyzed for mitochondrial activity by incubation with 26 nM JC-1 (T3168, Thermo Fisher) for 30 min at 38 °C in the dark. The evaluation was conducted using fluorescence microscopy (Leica^®^ DM4000 LED, Wetzlar, Germany, Ex./Em. 488/520 nm) simultaneously to transmit light brightfield at ×40 magnification. Two hundred spermatozoa per sample were classified as having a high (orange fluorescence) or a low (green fluorescence) mitochondrial membrane potential. The Mitochondrial Activity parameter was expressed as the percentage of spermatozoa with high mitochondrial activity per sample.

#### 2.5.5. Sperm DNA Fragmentation

The DNA fragmentation was analyzed with the Halomax^®^ kit (Halotech^®^ DNA, Madrid, Spain). First, the agarose was heated to 100 °C for 5 min and then allowed to cool down to 37 °C for 5 min. Subsequently, the sperm samples were added to the agarose gel (1:2, *v*/*v*) and thoroughly mixed. A 2 µL drop of the mixture was placed onto a slide, covered with a glass coverslip, and left at 4 °C for 10 min to solidify. The coverslip was removed, and the samples were treated with the first lysis solution for 5 min, followed by distilled water for an additional 5 min. Finally, the slides were dehydrated with sequential 70% and 100% ethanol and stained with a fluorescent red stain (Fluored^®^ HT-RFS100, Halotech DNA, Madrid, Spain). The spermatozoa were then evaluated under fluorescence microscopy at ×40 magnification (Leica^®^ DM4000 LED, Wetzlar, Germany, Ex./Em. 495/520 nm). Two hundred spermatozoa per sample were classified as unfragmented DNA (small and compact halo of chromatin dispersion) or fragmented DNA (large and spotty halo of chromatin dispersion). The DNA Fragmentation parameter was expressed as the percentage of DNA-fragmented spermatozoa per sample.

### 2.6. Artificial Insemination (AI) and Pregnancy Diagnosis

Estrus detection of sows was conducted daily in the presence of a sexually mature boar starting on the day of weaning. Sows in heat were inseminated with AI doses by post-cervical AI at estrus onset and 24 h later with the same boar. The AI was carried out right after the female stimulation with the boar’s presence and nose-to-nose contact. Fertility parameters were monitored through return-to-estrus starting on day 18 after the AI. Sows exhibiting estrus signs were considered non-pregnant. Furthermore, pregnancy was diagnosed via ultrasound 23–28 days after AI using transabdominal ultrasound (Echoscan T-300 S, Barcelona, Spain). The sows diagnosed as pregnant were housed in collective pens with 8–10 sows per pen.

### 2.7. Farrowing, Litter Performance, and Offspring Growth

Pregnant sows were transferred from gestation facilities to the farrowing crates at 110 days of gestation. At the time of farrowing, the parameters recorded were as follows: Farrowing Rate (%), as the percentage of sows that successfully gave birth out of the total number of sows that were inseminated; the Parity, as the number of farrowings of each sow; the Gestation Length (days), as the number of days from the day of insemination to the day of farrowing per sow; the Total Born, which includes all piglets that were born per sow independently of the state at birth (alive, stillborn, or mummified); the Born Alive, as the number of live piglets born per litter; the Stillborn, as the number of dead piglets born per litter; the Mummified, as the number of mummified piglets born per litter; the Males, as the number of total males born per litter; the Females, as the number of total females born per litter; the Sex Ratio (females/males), as the number of total females per total males born per litter; the Males Born Alive, as the number of live male piglets born per litter; the Females Born Alive, as the number of live female piglets born per litter; the Alive 24 h, as the number of live piglets after 24 h of birth per litter; the Dead 24 h, as the number of dead piglets after 24 h of birth per litter; the Males Alive 24 h, as the number of live male piglets after 24 h of birth per litter; the Females Alive 24 h, as the number of live female piglets after 24 h of birth per litter; the Piglet Weight 24 h (kg), as the weight per live piglet after 24 h of birth; the Litter Weight 24 h (kg), as the weight per litter after 24 h of birth. A precision scale (ZMISSIL F1-30; Gram Precision S.L., Hospitalet de Llobregat, Spain) was used to measure the body weight (kg).

### 2.8. Blood Collection and Analysis

Blood samples were collected 7 days after the piglets’ birth. The timing for blood collection was chosen to avoid excessive handling of newborns within the first hours after birth and ensure that fluctuation in physiological parameters related to stress after birth was stable and did not interfere with the results. The blood extraction was carried out via venipuncture of the jugular vein using a Vacutainer system (BD Vacutainer^®^ 21G 0.8 × 25 mm needle; BD Vacutainer, Beckton and Dickinson España S.A., Madrid, Spain) and lithium heparin tubes. Approximately 4–5 mL of blood was collected per piglet. The blood samples were transported to the laboratory within 1 h in a Styrofoam box and kept refrigerated at 4 °C until analysis, which was performed within 18 h of collection. Hematological analysis was conducted using an ADVIA© 120 analyzer (Siemens, Holliston, MA, USA), and the biochemical serum parameters were evaluated using Olympus AU600 (Olympus Co., Tokio, Japan) and Mindray BS-200E (Mindray, Nanshan, Shenzhen, China) analyzers.

The biochemical serum parameters determined per blood sample were the concentration of Total Protein (g/dL), Albumin (g/dL), Globulins (g/dL), Creatinine (mg/dL), Urea (mg/dL), Glucose (mg/dL), Cholesterol (mg/dL), Triglycerides (mg/dL), Amylase (UI/L), Lipase (UI/L), Creatine Kinase (CK, UI/L), Alkaline Phosphatase (ALP, UI/L), γ-Glutamyl Transferase (GGT, UI/L), Aspartate Aminotransferase (AST, UI/L), Alanine Aminotransferase (ALT, UI/L), Bilirubin (mg/dL), Calcium (mg/dL), Potassium (mmol/L), Sodium (mmol/L), and Chlorine (mmol/L).

The hematological parameters were as follows: the Hemolysis (1–3), as the presence of hemolysis by visualization in a scale 0 to 3 (0 indicates no hemolysis, 1 almost nil, 2 moderate, and 3 severe hemolysis); Erythrocytes Concentration (×10^6^ cells/µL); Hematocrit (%); Hemoglobin Concentration (g/dL); Mean Corpuscular Volume (MCV, fL); Mean Corpuscular Hemoglobin (MCH, pg); Mean Corpuscular Hemoglobin Concentration (MCHC, g/dL); Cell Hemoglobin Concentration Mean (CHCM, g/dL); Erythrocyte Distribution Width (RDW, %); Cell Hemoglobin Content (CHC, pg); Cell Hemoglobin Distribution Width (CHDW, pg); Hemoglobin Distribution Width (HDW, g/dL); Leukocytes Concentration (×10^3^ cells/µL); Neutrophils Concentration and relative proportion in Leukocytes (×10^3^ cells/µL and %); Lymphocytes Concentration and relative proportion in Leukocytes (×10^3^ cells/µL and %); Monocytes Concentration and relative proportion in Leukocytes (×10^3^ cells/µL and %); Eosinophils Concentration and relative proportion in Leukocytes (×10^3^ cells/µL and %); Basophils Concentration and relative proportion in Leukocytes (×10^3^ cells/µL and %); Platelets Concentration (×10^3^ cells/µL); Plateletcrit (%); Mean Platelet Volume (MPV, fL); Platelet Distribution Width (PDW, %); Mean Platelet Component (MPC, g/dL); Platelet Component Distribution Width (PCDW, g/dL); Mean Platelet Mass (MPM, pg); Platelet Mass Distribution Width (PMDW, pg); Large Platelets Concentration (×10^3^ cells/µL); Reticulocytes Concentration and relative proportion in blood cells (×10^6^ cells/µL and %); Reticulocyte Hemoglobin Content (CHr, pg); Reticulocyte Mean Corpuscular Volume (MCVr, fL).

### 2.9. Experimental Design

**Figure 1:**
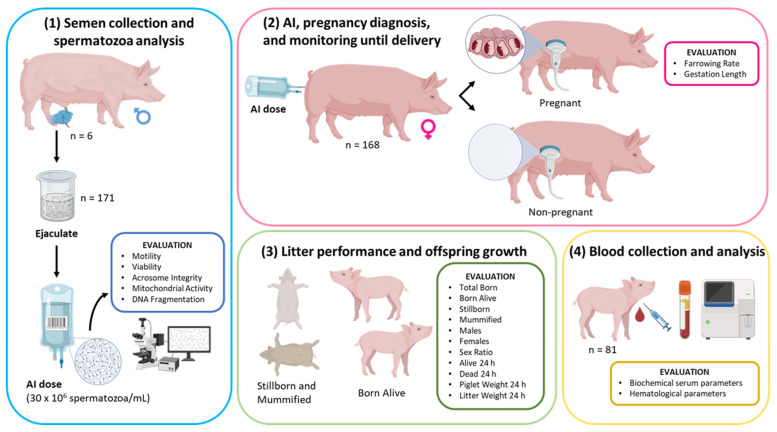
Graphic scheme of the experimental design. (1) Ejaculates (n = 171) from different boars (n = 6) were obtained and prepared as homospermic AI doses. Sperm parameters evaluated per AI dose: Spermatozoa Motility, Viability, Acrosome Integrity, Mitochondrial Activity, and DNA Fragmentation. (2) Multiparous sows (n = 168) were inseminated with the homospermic AI doses and monitored for pregnancy diagnosis by ultrasound and until delivery. The data about Gestation Length per sow and Farrowing Rate were registered. (3) After farrowing, the litters were evaluated and the parameters registered were the Total Born, Born Alive, Stillborn, Mummified, Males, Females, Sex Ratio, Males Born Alive, Females Born Alive, Alive 24 h, Dead 24 h, Males Alive 24 h, Females Alive 24 h, Piglet Weight 24 h, and Litter Weight 24 h. (4) A representative set of piglets (n = 81) were selected randomly and equally distributed from litters and were subjected to blood collection and analysis of biochemical serum and hematological parameters (see Section 2.8). Created in Biorender (https://BioRender.com, accessed on 1 May 2025).

The present study aimed to determine whether boars with high fertility and prolificacy exhibited sperm parameters that could be associated with the productivity and health of their offspring. For that purpose, the ejaculates from independent boars were obtained to prepare homospermic AI doses to inseminate sows and analyze the descendants. Thus, the experiment was conducted as follows (Figure 1):

In the first step, 171 ejaculates were obtained from 6 boars in a balanced number of ejaculates per boar. The ejaculates were analyzed for volume and concentration. After that, sets of homospermic AI doses were prepared from each ejaculate and examined for the spermatozoa parameters described above.

Secondly, 168 sows were inseminated with the AI doses in a balanced number of sows per boar and monitored for pregnancy diagnosis, gestation, and until delivery. The parameters registered were described in the section for farrowing, litter performance, and offspring growth analysis.

Finally, 81 piglets were selected randomly within the litter in a balanced proportion by sex (41 females and 40 males) and equally distributed from the litters obtained. The piglets were subjected to blood collection and analysis as described in the corresponding section.

### 2.10. Statistical Analysis

Data analyses were conducted in the R statistical environment v. 4.4 [8]. Associations among variables within and between each data set (sperm, litter, and blood parameters) were established by Pearson correlations. The effects of the sperm variables on the litter and blood parameters were further analyzed by multiple linear regression, using uncorrelated variables as predictors and a stepwise method based on AIC (Akaike Information Criteria) optimization to remove or add variables.

The relationships between the sperm variables (independent variable set) and the litter characteristics and blood analysis parameters (dependent variables sets) were determined using Canonical Correlation Analysis (CCA). The CCA yields two sets of canonical variates for each determination, producing indices enabling the study of the relationship between the original variables and the new canonical variates (loadings and cross-loadings). Complementarily, we used MANOVA and Canonical Discriminant Analysis (CDA) to test the influence of the boar in each set of litter and blood parameters.

The significance threshold was adjusted at *p* ≤ 0.05 in all cases, using the false discovery rate for adjusting the *p*-values in multiple testing. In the case of the multiple linear regression, models were accepted with *p* < 0.1.

## 3. Results

### 3.1. Descriptive Statistics and Within and Between Variable Set Correlation

The descriptive statistics for the ejaculates and AI doses (sperm), the litters, and the blood parameters of the piglets are shown in Supplementary Material Tables S1–S4. The parameters analyzed in the ejaculates and spermatozoa were considered to be in the normal range for selected and highly fertile boars from commercial farms. To name a few, the median of Total Motility was 92%, with a Progressive Motility of 35%. The median of the Viability obtained was 92%, with a 95% Acrosome Integrity (intact acrosomes). The DNA Fragmentation showed a wide range, between a minimum of 0% and a maximum of 9% (Table S1). The sows, also selected for their fertility and prolificity, were in a median of their 4th parity. They carried out a median Gestation Length of 116 days, with a week difference between the minimum of 112 and the maximum of 119 days. Of the 168 sows inseminated, 152 sows became pregnant, carried to term, and gave birth to a litter, which meant a Farrowing Rate of 90.47%. All the parameters related to the litter were considered in the normal range for commercial hyperprolific sows (Table S2). The biochemical and hematological parameters from 24 h old piglets were mainly in the normal range for recently born pigs in commercial farms. Nevertheless, a few parameters showed a noteworthy high variability, such as some pancreatic, hepatic, white blood cell series, and platelet parameters (Tables S3 and S4).

The association study among variables within each set (sperm, litter, and blood sets of variables) showed multiple highly significant correlations (Supplementary Material Tables S5–S7). This was unsurprising since many variables are derived or narrowly related, especially within CASA and the litter sets. The blood variables were also associated between them in a high proportion of the cases, notably the Sodium and Chlorine concentrations, which were negatively associated with the activity of several enzymes, except for the GGT. Additionally, the variables associated with the platelets correlated with most of the variables. The Plateletcrit was negatively associated with variables related to erythrocytes and corpuscular hemoglobin. The presence of Large Platelets was positively associated with the activity of GGT and negatively with AST and ALT.

Contrarily, the correlations between variable groups were small and scarcely significant (Supplementary Material Tables S8–S10). Interestingly, ALH correlated negatively with the Gestation Length (−0.305, *p* = 0.019) and positively with the Stillborn (0.282, *p* = 0.042), and BCF negatively with the Gestation Length (−0.353, *p* = 0.002), suggesting that vigorous motility could be related to a shorter pregnancy (Table S8). Considering the blood parameters, we only found a significant correlation with the CASA parameters (Table S9) for VAP, which was negatively correlated with Calcium concentration in blood (−0.440, *p* = 0.038), with no significant correlations with the litter parameters (Table S10).

### 3.2. Multivariate Linear Regression of Sperm Variables for Explaining Litter and Blood Parameters

The multivariate linear regression analysis used a stepwise algorithm (AIC optimization) starting with a reduced set of uncorrelated variables (otherwise, models were unsolvable): Ejaculate Volume, Progressive Motility, VCL, Viability, and DNA Fragmentation. It yielded potentially useful models (*p* < 0.1; Table 1 and Table 2), with some consistently selected CASA variables. Whereas the explanatory power of the models (as R^2^) was very low in all cases (below 0.139 in Table 1 and 0.23 in Table 2), these variables showed potential for influencing litter and especially the piglet blood parameters. The most remarkable effect of the sperm parameters on the litter (Table 1) came from the DNA Fragmentation of spermatozoa, which showed a significant impact both on the Piglet Weight 24 h (*p* = 0.008) and Litter Weight 24 h (*p* = 0.005). Regarding the effect of sperm parameters on the piglets’ blood parameters (Table 2), the DNA Fragmentation had a significant effect on the Hemolysis (*p* = 0.006), the Ejaculate Volume and Viability on the blood Glucose levels (*p* < 0.001 and *p* = 0.009, respectively), and Progressive Motility on blood Lipase concentrations (*p* = 0.003). Progressive Motility also showed a significant effect on several hematological parameters, especially on cells of the white blood cell series, such as Leukocytes (*p* = 0.016), Monocytes (*p* = 0.022), and Basophils Concentrations (*p* = 0.004). VCL had a significant effect on the HDW (*p* = 0.008), Leukocytes Concentration (*p* = 0.005), and the Ejaculate Volume and Viability on the Basophil proportion (*p* = 0.009 and *p* = 0.004, respectively).

### 3.3. Canonical Correlation Analysis (CCA) of the Sperm Variable Set with the Litter and Blood Variables Sets

CCA determined the multivariate relationship between the sperm parameters and the litter and blood parameters. The CCA with the sperm variables (explanatory, X-set) and the litter variables (response, Y-set) in the CCA yielded only a significant canonical correlation (X_1_Y_1_; Table 3), accounting for 44.5% of the variability in the relationship between both sets. While the X_1_Y_1_ canonical correlation was significant (*p* = 0.020), it showed a low coefficient (Canonical R of 0.396). The canonical loadings (Table 4), a measure of the contribution of the individual starting variables to the canonical variates X_n_ and Y_n_, showed that X_1_ was remarkably and positively related to Progressive Motility (X_1_ = 0.86) and negatively to VCL (X_1_ = −0.89), Viability (X_1_ = −0.59), and Mitochondrial Activity (X_1_ = −0.71); Y_1_ was mainly positively related to Dead 24 h (Y_1_ = 0.63) and negatively to Stillborn (Y_1_ = −0.69). The Supplementary Material shows additional information on the CCA such as the standardized canonical coefficients (Table S11) and the cross-loadings (Table S12). The standardized canonical coefficients show the relative contribution of each initial variable to the variate (the CCA produces an optimal linear combination of the initial variables); the cross-loadings show the correlations of each initial variable set to the canonical variate resulting from the other variable set.

Nevertheless, the CCA between the sperm variable set and the blood variable set did not produce any significant canonical correlation. Despite a high R (Canonical R > 0.9) due to the number of variables involved, the corresponding *p*-value was higher than 0.8.

### 3.4. Canonical Discriminant Analysis (CDA) of the Sperm, Litter, and Blood Variables Sets for the Boar Factor

The CDA studied the effects of the boar on the variates obtained after a multivariate analysis of each variable set (MANOVA). Table 5 shows the characteristics of the resulting two first variates (Can1 and Can2) for each variable set (sperm, litter, and blood parameters sets). Only Can 1 was significant for the sperm variables set (Can1: *p* < 0.001), accounting for 61.3% of the variability and R = 0.482. No significant variates resulted from the litter parameters (Can1: *p* = 0.090; Can2: *p* = 0.247). Both Can1 and Can2 were significant for the blood variable set (Can1: *p* < 0.001; Can2: *p* = 0.007), accounting for a cumulative of 54.7% and R = 0.755 and 0.747, respectively.

**Figure 2:**
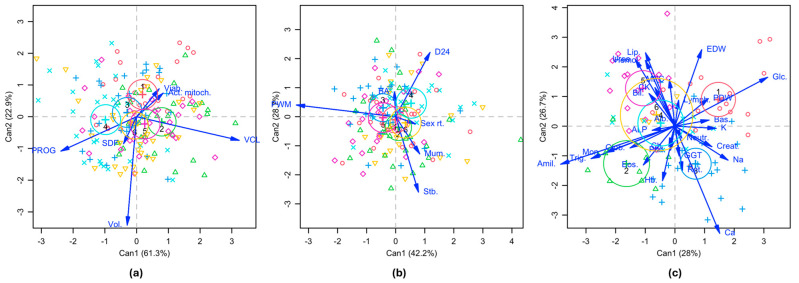
Graphical representation of the two first canonical dimensions (Can1 and Can2) from the Canonical Discriminant Analysis (CDA) performed on the sperm (**a**), litter (**b**), and blood (**c**) parameters for the boar effect. The plots show the observations in the bidimensional space defined by Can1 and Can2, with the original variables as vectors defined by the canonical structure coefficients. The canonical means for each boar are plotted as crosses and labeled, with circles showing 95% confidence intervals. Vol.: Ejaculate Volume; PROG: Progressive Motility; VCL: Curvilinear Velocity; Viab.: Viability; Act. mitoch.: Mitochondrial Activity; SDF: DNA Fragmentation; Stb.: Stillborn; Mum.: Mummified; D24: Dead 24 h; PWM: Piglets Weight 24 h; Sex rt.: Sex Ratio (females/males); Hemol: Hemolysis; Alb.: Albumin; Glob.: Globulins; Creat.: Creatinine; Urea: Urea; Glc.: Glucose; Ch.: Cholesterol; Trig.: Triglycerides; Amil.: Amylase; Lip.: Lipase; CK: Creatinine Kinase; ALP: Alkaline Phosphatase; GGT: γ-Glutamyl Transferase; Bil.: Bilirubin; Ca: Calcium; K: Potassium; Na: Sodium; Hb.: Hemoglobin; EDW: Erythrocyte Distribution Width; Neutr.: Neutrophils (%); Lymph.: Lymphocytes (%); Mon.: Monocytes (%); Eos.: Eosinophils (%); Bas.: Basophils (%); Plat.: Platelets; PDW: Platelet Distribution Width; MPC: Mean Platelet Component; Ret.: Reticulocytes (%). The observations of the different males are represented as follows: boar 1, light pink circles; boar 2, green triangles; boar 3, dark blue crosses; boar 4, light blue equis; boar 5, light pink diamonds; boar 6, yellow inverted triangles.

Figure 2 shows bivariate plots for each CDA of each variable set, including the observations, vectors for the variables included in each analysis, and the influence of the boar as a factor. The plots include the first two variates (Can1 and Can2) for homogeneity, irrespective of their significance. The details for each variate (standardized canonical coefficients and canonical structure coefficients) are shown in the Supplementary Material Tables S13–S18. The most remarkable canonical structure coefficients for the sperm variables set (Figure 2a and Table S14) showed that Can1 was positively influenced by VCL (0.808) and negatively by Progressive Motility (−0.597) and Can2 negatively by Ejaculate Volume (−0.854). For the litter variables (Figure 2b and Table S16), Can1 was positively influenced by Dead 24 h (0.311), Mummified (0.218), and Stillborn (0.207) and negatively by Piglet Weight 24 h (−0.899). Can2 was positively influenced by Dead 24 h (0.582) and negatively by Stillborn (−0.683) and Mummified (−0.337). For the blood variables (Figure 2c and Table S18), it was remarkable that Can1 was positively influenced by Glucose (0.447) and negatively by Amylase (−0.550), Triglycerides (−0.404), and Monocytes Concentration (−0.333). Can2 was positively related to Hemolysis (0.316), Urea (0.322), Lipase (0.354), and EDW (0.368) and negatively to Calcium (−0.516).

As suggested by the CDA results, the plots for the canonical structure coefficients in Figure 2a–c showed that the boars (1 to 6, color circles) were closely clustered for the analyses regarding the sperm parameters (only Can1 was significant, Table 5) and litter variables (no variate reaching statistical significance). For blood parameters, the influence of the boar was evident (both Can1 and Can2 were significant, Table 5). Attending to the distribution of the observations, for the sperm variables analysis (Figure 2a), one of the boars (num. 4) visibly separated for Can1 with the stronger effect of the Progressive Motility and VCL. The boar 4 cases (light blue equis), with respect to the central vertical line of Can1, were distributed in the same direction as the vector representation of the Progressive Motility and in the opposite direction to the vector representation of VCL. The other five boars (num. 1, 2, 3, 5, and 6) clustered together, with more homogeneous results. However, for the blood variables analysis (Figure 2c), four of the boars (boars 1, 2, 3, and 5) were clearly separated with respect to either the central vertical line of Can1 or the horizontal line of Can2. The Can1 was explained by boars 1 (light pink circles) and 3 (dark blue crosses), in which the most influenced blood parameters in the progeny were the Erythrocyte Distribution Width (EDW), and the Glucose (Glc.) for boar 1, and mainly the serum Calcium (Ca) for boar 3. Can1 was also explained by boars 2 (green triangles) and 5 (light pink diamonds), in which the most influenced blood parameters in the progeny were the Amylase (Amil.), Triglycerides (Trig.), Monocytes (Mon.), and Globulins (Glob.) for boar 2 and Lipase (Lip.), Urea (Urea) and Hemolysis (Hemol.) for boar 5. On the other hand, the influence of the boar on their progeny blood parameters for Can2 clearly separated males 1 and 5 from males 2 and 3, with the primary influence of the same variables described for Can1. In contrast, two overlapped (boars 6 and 4) and had a lower influence on the variables described since they were close to the central cross of the vertical line of Can1 and the horizontal line of Can2. Nevertheless, boar 6 showed a higher data dispersion than the other boars, plotted as a wider yellow circle.

## 4. Discussion

Selection of high-quality semen from genetically superior boars is crucial for achieving optimal fertilization rates and passing desirable traits to offspring, such as improved growth efficiency, disease resistance, and optimal reproductive performance [9]. However, minimal variation among high-quality boars can further maximize farm productivity, highlighting the importance of selecting boars with exceptional reproductive traits while maintaining animal welfare and productivity standards [9]. Despite the impact on farm performance, the specific influence of individual boars and certain sperm characteristics on offspring health and productivity remains underexplored in livestock production. This study investigated how sperm from individual boars affects specific parameters in their progeny after AI of sows on a commercial farm. The observational design of the study offers both advantages and limitations. It objectively assesses the boar’s impact under commercial conditions and contributes valuable new data to the field. In general terms, our model’s predictive values (R^2^ values) are low when assessing the individual effect of each variable. However, many of these effects are statistically significant. This suggests that while several paternal sperm parameters are associated with productive and blood indicators, none alone exerts a strong enough influence on offspring traits. Instead, the combined interaction of multiple parameters within a complex biological interplay of multiple factors likely drives the overall paternal effect, as reviewed by other authors [10]. Given this scenario, it seems more logical to think that multivariate analyses can be models that, although with limitations, better adapt to nature.

Since maternal influences during pregnancy and lactation also play a significant role and are a source of variation [11], separating paternal and maternal effects is challenging due to shared genetic and environmental factors. As with multiple other studies, this work effectively isolated the paternal effect by controlling or standardizing maternal variables [12,13]. This allowed a more accurate assessment of how individual boars influence piglet indicators. Nevertheless, some maternal parameters affecting the offspring were also evaluated, such as the gestation length. This study revealed a significant male effect on the gestation length in sows inseminated with high-performing boars, with differences of up to one week. Such variability can have substantial economic implications for pig production, as longer gestation can disrupt synchronized farrowing schedules [14], leading to increased labor costs, inefficient facility uses, and challenges in managing piglet care and weaning. Conversely, shorter gestations may result in an increased incidence of piglets with low birth weight and higher early mortality rates [15]. Thus, sire-related variability in gestation length must be optimized to ensure optimal herd productivity and profitability. This is because such variability can lead to increased feed and maintenance costs for sows and potential losses due to lower piglet survival and growth [16]. As reported earlier, the male effect may be attributed to genetic differences affecting sperm quality, such as motility, morphology, viability, or DNA integrity [17]. These factors are essential for successful fertilization, early embryonic development, embryo implantation [18], and, ultimately, the health and viability of offspring [19]. Sperm viability can shape key metabolic and stress-response pathways in developing piglets [20], with these effects potentially measurable in early life blood parameters most probably related to the glycolytic metabolism, oxidative stress, and other epigenetic and proteomic influences, which remain to be more deeply investigated. In addition, it has been demonstrated that seminal plasma components can influence gestation length by altering the maternal uterine environment, thereby affecting embryo–maternal communication and implantation success [6,21]. To mitigate these effects, swine producers should adopt advanced boar selection strategies prioritizing reproductive performance at pregnancy rates and beyond. Implementing genetic evaluations and targeted breeding programs can help reduce gestation variability and optimize time. Further research into the biological mechanisms behind the male effect on gestation is crucial for developing effective strategies to enhance reproductive efficiency.

This study also shed light on the individual effect of the boar sperm motion in the progeny, especially parameters related to sperm velocity, progressivity, and vigorous movement. Sperm motility reflects the ability of this cell to navigate the female reproductive tract and fertilize the oocyte, improving embryo viability and uniform development [22]. In particular, sperm velocity and progressive motility have been identified as vital indicators of sperm functionality and fertility [23]. High sperm velocity and progressivity suggest strong mitochondrial function and energy production, both essential for successful fertilization [24,25] and even beyond fertilization [26]. It has been suggested that embryo development could be tightly associated with glycolysis-related metabolites derived from the energetic status of the paternal spermatozoa [20], which is usually reflected in their progressive motility and velocity. In this respect, the lack of association in the linear regression of paternal sperm mitochondrial activity and piglets’ blood indicators in our results suggest that this association is complex and probably indirect and/or non-linear. While sperm mitochondria are typically degraded post-fertilization, their functional status prior to this event can significantly influence early embryonic development, yet excessive activity may result in oxidative stress and DNA damage, affecting embryo quality and, most probably, postnatal health. The influence of mitochondrial function on offspring phenotypes may most probably be mediated through interactions with other cellular processes. Further research is necessary to shed light on this respect.

Our study revealed a significant relationship between sperm motility and gestational length. ALH and BCF, indicators of hyperactivated-like and vigorous motility, respectively, showed a negative effect on the gestation length, suggesting that zestful sperm motility could be associated with shorter gestation periods and bringing to light the complex interaction between sperm quality and gestational timing. As published earlier in several species, enhanced motility ensures that the most functionally competent sperm reach the oocyte [27]. These spermatozoa have been linked to lower chances of early embryonic loss [28] and lead to efficient placental development [29], which could potentially be linked to an optimal gestational length. On the other hand, it is crucial to consider the role of other components of the semen that modulate sperm functionality and have demonstrated effects on sperm motility and uterine environment, such as the seminal plasma [30]. In summary, the results of the present work emphasize the importance of incorporating advanced sperm motility assessments into boar selection criteria to improve reproductive efficiency.

The ejaculate volume is a key determinant of sperm output. It is often associated with the health of accessory sex glands and reproductive performance (reviewed by [31]). Larger ejaculate volumes typically imply a higher total sperm count, increasing the likelihood of successful fertilization. However, our study suggests that this relationship is not purely quantitative. Individual variation among boars affects that relationship, and higher ejaculate volume does not uniformly translate to better reproductive outcomes. Several physiological factors could explain this variation. Boars with larger ejaculate volumes might differ in accessory gland function or seminal plasma composition, potentially affecting sperm transport and fertilization efficiency (reviewed by [32]). Differences in these components could account for the observed individual effects, where some boars with larger ejaculates produce more viable or superior-performing offspring while others do not. The boar ejaculate volume is also linked to different seminal plasma compositions, which could condition the metabolic and oxidative pathways in the developing piglet, potentially evidenced by distinct early life blood parameters. Extensive studies in boar selection programs could elucidate these complex interactions.

We found an impact of DNA fragmentation on the piglet weight (as total and averaged by piglet), revealing the impact of paternal genetic integrity on neonatal outcomes and early offspring development. Mechanistically, fragmented DNA can lead to faulty or incomplete gene expression during critical stages of embryogenesis and has been linked to compromised embryonic development in various species, including pigs [33]. This phenomenon could be partially attributed to placental inefficiencies caused by compromised embryonic development linked to paternal DNA damage. As demonstrated in ungulates, the consequence would be a litter composed of underdeveloped piglets, lowering the overall litter weight and increasing the risk of neonatal mortality and poor postnatal growth performance [34]. Sperm DNA fragmentation in boars may influence biochemical pathways in piglets through epigenetic alterations and oxidative stress mechanisms, potentially manifesting in blood indicators at early ages, but the exact mechanisms and pathways involved still need to be elucidated.

Recent studies in humans reported a positive correlation of sperm DNA fragmentation with miscarriage rates and a negative correlation with lower birth weight of children conceived by assisted reproductive technology [35]. Our results also showed that the boars significantly influenced early piglets’ mortality after birth and the proportion of stillborn. Traditionally, maternal factors such as uterine environment and nutrition have been emphasized as primary determinants of fetal growth. However, the findings here reinforce the growing understanding that paternal genetic quality, particularly sperm DNA integrity, plays a substantial role in early developmental outcomes. The observed associations underscore the necessity of elucidating the underlying causes of DNA fragmentation in boar sperm. Studies in humans [36] have revealed that environmental stressors, including heat stress, poor nutrition, and exposure to toxins, can elevate oxidative stress, increasing DNA damage. While such studies are still scarce in swine, there is evidence that similar insults could affect sperm DNA fragmentation [37,38,39]. Therefore, monitoring sperm DNA quality in boars and selecting individuals with low DNA fragmentation becomes a reproductive concern and a matter of economic significance in swine production systems.

It is well established that hematological traits, such as erythrocyte count and hemoglobin concentration are heritable in humans [40]. Boars with superior genetic profiles, which facilitate efficient oxygen transport, may transmit these advantages to their offspring. The study’s findings likely reveal significant variations in erythrocyte and hemoglobin levels among piglets sired by different boars, suggesting that paternal genetics play a crucial role in these hematological parameters. Higher erythrocyte counts and hemoglobin levels enhance oxygen delivery to tissues, supporting better survival and growth rates in piglets [41]. This also aligns with the importance of adequate oxygen supply for optimal cell metabolism and organ function [42,43]. These findings could have relevant repercussions because if particular boars consistently influence hematological metrics in their piglets, then the selection of boars could help to optimize growth performance and survivability.

This study presented boar-derived variates that influenced the cellular component of the descendants’ blood. The red cell distribution width (RDW) measures the variability in erythrocyte size, reflecting erythropoiesis and bone marrow function. The observed boar effect on piglet RDW suggests a possible paternal influx in regulating the descendants’ hematopoiesis. This is consistent with previous studies that found upregulated genes related to hematopoiesis in high-fertility boars [44]. On the other hand, the parameters for the white blood cell (WBC) series serve as critical indicators of immune competence, and this study revealed a significant paternal influence on the WBC series in piglets. In this sense, the seminal plasma could also participate [45,46,47,48] and should not be discarded, but it still needs to be probed. Consequently, piglets from these boars could potentially develop robust immune systems and exhibit better growth performance. Furthermore, variations in boar sperm quality were also linked to changes in platelet levels, which would influence coagulation homeostasis [49,50]. This suggests that boars with superior sperm characteristics may transmit genetic or epigenetic advantages that enhance hematopoiesis in their offspring.

Differences in piglets’ blood markers related to vital organs’ functions also seemed to be strongly conditioned by the individual paternal effect on the descendants. This would be the case for the pancreas (lipase), liver (alanine and aspartate aminotransferases, ALT and AST, respectively), and kidney (urea) functions, glucose homeostasis, lipid metabolism (triglycerides), oxidative stress (bilirubin), protein catabolism (urea), carbohydrate breakdown (amylase), and muscle contraction and neural signaling (calcium). DNA integrity and motility, in particular, appear to be primarily associated with these parameters. A tentative explanation is that DNA integrity and motility critically influence embryonic development and, therefore, the subsequent formation of organs such as the pancreas, liver, and kidney, which sustain core functions in metabolism [51]. This fact also means that individual boars with normal “standard” sperm parameters but altered “advanced” sperm indicators may go unnoticed. Such boars may contribute to a decrease in farm productivity and an increased susceptibility of the piglets to metabolic disorders, early mortality, or disease. Boars selected by transmitting better traits related to physiological functions as efficient erythropoiesis, and oxygen transport capacity can produce progeny with more stable glucose levels, improved growth rates, and enhanced resilience against hypoglycemia, a prevalent cause of neonatal mortality in piglets. These boars can also contribute to balanced lipid metabolism, robust and well-regulated immune systems, and improved overall herd health and productivity. Sperm indicators should be considered to detect specific boars that enhance productivity or, on the contrary, which can be discarded to maintain farm standards. A strategy for boars’ selection based on predictive indicators of productivity can lead to optimized gestation length and synchronized births, reduced veterinary costs, and ultimately enhanced productivity in swine operations, contributing to the development of more sustainable and profitable livestock management practices. While the multivariate selection model proposed in this study aims to enhance productivity by identifying optimal paternal sperm traits, it is essential to acknowledge the intense selection practices in current pig production, even in meat quality. These considerations highlight the need for balanced breeding strategies incorporating performance and health-related traits to ensure sustainable pig production systems.

## 5. Conclusions

The multiple parameters analyzed using a multivariate strategy enabled an integrated and more complete assessment of the repercussions of boar sperm status and functionality on litter characteristics, including hematological parameters. Our study evidenced that slight variations between high-quality boars could affect the offspring characteristics despite the high homogeneity of sperm quality analyzed in farms. Some specific semen variables could significantly affect the primary physiological and productive parameters of their descendants, and this work highlights the critical role of boar sperm quality in shaping essential functions in offspring.

Incorporating advanced sperm evaluation techniques, e.g., flow cytometry for assessing viability and DNA fragmentation, computerized motility analysis, and multidimensional selection criteria in pig breeding programs, can improve boars’ selection, transmitting superior or desirable characters to the descendants. Future studies should investigate the molecular and genetic mechanisms underlying these relationships and assess how these hematological and metabolic advantages translate into long-term growth and productivity.

## Figures and Tables

**Table 1:** Significant linear multivariate models explaining litter parameters. Only the models with a low *p*-value are detailed.

Litter Parameter	Model Components (Sperm Analysis Variables)	Coefficient Estimate	Coefficient Standard Error	*p*-Value	Model Adjusted R^2^	Model *p*-Value
Total Born	Intercept	0.115	0.130	0.380	0.05	0.085
	Ejaculate Volume	−0.734	0.415	0.085		
Stillborn	Intercept	−0.077	0.145	0.600	0.07	0.043
	Viability	2.275	1.091	0.043		
Females	Intercept	−0.015	0.148	0.9193	0.09	0.093
	Ejaculate Volume	−1.047	0.456	0.0271		
	Progressive Motility	2.343	1.112	0.0416		
	VCL	1.041	0.609	0.0950		
	DNA Fragmentation	−3.451	2.473	0.1706		
Piglet Weight 24 h	Intercept	0.107	0.158	0.501	0.139	0.029
	Progressive Motility	2.484	1.176	0.041		
	VCL	1.340	0.647	0.045		
	DNA Fragmentation	−7.745	2.634	0.005		
Litter Weight 24 h	Intercept	−0.009	0.164	0.958	0.12	0.065
	Progressive Motility	2.118	1.223	0.091		
	VCL	1.353	0.778	0.090		
	Viability	−2.214	1.560	0.164		
	DNA Fragmentation	−7.778	2.800	0.008		

**Table 2:** Significant linear multivariate models explaining blood parameters. Only the models with a low *p*-value are detailed.

Blood Parameter	Model Components (Sperm Analysis Variables)	Coefficient Estimate	Coefficient Standard Error	*p*-Value	Model Adjusted R^2^	Model *p*-Value
Hemolysis	Intercept	0.207	0.140	0.146	0.15	0.013
	Ejaculate Volume	−0.916	0.449	0.048		
	DNA Fragmentation	3.810	1.317	0.006		
Globulins	Intercept	−0.059	0.133	0.659	0.12	0.025
	Ejaculate Volume	1.020	0.427	0.022		
	DNA Fragmentation	−2.780	1.253	0.032		
Urea	Intercept	−0.008	0.123	0.948	0.06	0.065
	Viability	1.756	0.926	0.065		
Glucose	Intercept	−0.108	0.121	0.377	0.22	0.002
	Ejaculate Volume	−1.865	0.504	<0.001		
	Viability	−3.236	1.176	0.009		
Triglycerides	Intercept	0.072	0.115	0.537	0.05	0.079
	Ejaculate volume	0.664	0.368	0.079		
Lipase	Intercept	−0.018	0.121	0.881	0.16	0.011
	Progressive motility	1.414	0.446	0.003		
	Viability	2.244	1.086	0.045		
Creatin Kinase (CK)	Intercept	−0.038	0.140	0.789	0.05	0.083
	Viability	1.877	1.056	0.083		
Alanine Aminotransaminase (ALT)	Intercept	0.019	0.143	0.893	0.06	0.058
	Viability	2.093	1.075	0.058		
Calcium	Intercept	0.107	0.158	0.501	0.139	0.029
	Progressive Motility	2.484	1.176	0.041		
	VCL	1.340	0.647	0.045		
Potassium	Intercept	−0.191	0.168	0.263	0.08	0.071
	VCL	0.580	0.380	0.134		
	DNA Fragmentation	−2.420	1.500	0.114		
Sodium	Intercept	−0.197	0.150	0.196	0.06	0.063
	DNA Fragmentation	−2.561	1.340	0.063		
Chlorine	Intercept	−0.198	0.147	0.185	0.07	0.043
	DNA Fragmentation	−2.738	1.312	0.043		
Erythrocytes Concentration	Intercept	0.000	0.141	0.999	0.06	0.064
	DNA fragmentation	−2.400	1.262	0.064		
Cell Hemoglobin Concentration Mean (CHCM)	Intercept	1.116	0.138	0.999	0.06	0.053
	DNA Fragmentation	2.464	1.235	0.064		
Hemoglobin Distribution Width (HDW)	Intercept	−0.011	0.123	0.929	0.18	0.007
	VCL	1.075	0.388	0.008		
	Viability	−3.968	1.220	0.002		
Leukocytes Concentration	Intercept	0.031	0.126	0.805	0.14	0.016
	Progressive Motility	−1.327	0.528	0.016		
	VCL	−1.207	0.408	0.005		
Lymphocytes Concentration	Intercept	−0.022	0.126	0.864	0.08	0.067
	Ejaculate Volume	−0.996	0.527	0.066		
	Viability	−2.894	1.229	0.023		
Monocytes (%)	Intercept	0.134	0.103	0.203	0.10	0.048
	Ejaculate Volume	0.566	0.348	0.112		
	Progressive Motility	−0.798	0.334	0.022		
Monocytes Concentration	Intercept	0.141	0.116	0.231	0.07	0.090
	Progressive Motility	−0.953	0.428	0.031		
	Viability	−1.593	1.040	0.133		
Eosinophils (%)	Intercept	0.144	0.113	0.212	0.05	0.080
	Ejaculate Volume	0.651	0.363	0.080		
Eosinophils Concentration	Intercept	0.107	0.115	0.358	0.04	0.096
	VCL	−0.470	0.276	0.096		
Basophils (%)	Intercept	0.081	0.130	0.533	0.13	0.046
	Ejaculate Volume	−0.928	0.515	0.079		
	Progressive Motility	−1.784	0.619	0.006		
	Viability	−2.026	1.379	0.150		
	DNA Fragmentation	3.604	1.671	0.037		
Basophils Concentration	Intercept	0.030	0.113	0.793	0.23	0.004
	Ejaculate Volume	−1.285	0.470	0.009		
	Progressive Motility	−1.250	0.414	0.004		
	Viability	−3.761	1.246	0.004		
Platelets Concentration	Intercept	0.028	0.115	0.812	0.09	0.052
	Ejaculate Volume	0.528	0.367	0.158		
	VCL	−0.516	0.272	0.065		
Plateletcrit	Intercept	−0.023	0.111	0.834	0.05	0.071
	VCL	−0.493	0.266	0.071		
Platelet Distribution Width (PDW)	Intercept	0.001	0.104	0.990	0.06	0.069
	Ejaculate Volume	−0.622	0.333	0.069		
Platelet Component Distribution Width (PCDW)	Intercept	−0.048	0.115	0.681	0.08	0.033
	Progressive Motility	−0.788	0.358	0.033		
Platelet Mass Distribution Width (PMDW)	Intercept	0.040	0.127	0.756	0.09	0.059
	Ejaculate Volume	−0.961	0.408	0.023		
	DNA Fragmentation	1.714	1.196	0.159		
Reticulocytes Concentration	Intercept	−0.086	0.142	0.548	0.09	0.081
	Progressive Motility	−0.972	0.589	0.107		
	VCL	−1.125	0.518	0.036		
	Viability	2.594	1.419	0.075		

**Table 3:** Summary results for the canonical correlation analysis for the sperm variable set and the litter variable set. The Wilks Lambda tests were used if canonical correlations in the current row and all that follow were zero. The first (significant) canonical correlation is shown.

Variate	Canonical R	Canonical R^2^	Eigenvalues	Percent	Cumulative Percent	Wilks Lambda	d.f.	*p*-Value
X_1_Y_1_	0.396	0.157	0.186	44.5	44.5	0.676	36	0.020

d.f. = degrees of freedom.

**Table 4:** Canonical loadings of the original variables with their canonical variates for the sperm and litter variable sets. Only canonical variates from the first (significant) canonical correlation are shown.

X Variate	X_1_	Y Variate	Y_1_
Ejaculate Volume	0.27	Born Alive	0.16
Progressive Motility	0.86	Stillborn	−0.69
VCL	−0.89	Mummified	−0.26
Viability	−0.59	Dead 24 h	0.63
Mitochondrial Activity	−0.71	Sex Ratio	0.03
DNA Fragmentation	0.03	Piglet Weight 24 h	0.07

**Table 5:** Summary results for the Canonical Discriminant Analysis (CDA) on the boar effects on the variable sets. The Wilks Lambda tests were used if canonical correlations in the current row and all that follow were zero. The first two canonical correlations are shown.

Variable Set	Variate	Canonical R	Canonical R^2^	Eigenvalues	Percent	Cumulative Percent	Wilks Lambda	d.f.	*p*-Value
Sperm parameters	Can1	0.482	0.233	0.303	61.3	61.3	0.639	30	<0.001
	Can2	0.319	0.102	0.113	22.9	84.2	0.833	20	0.069
Litter parameters	Can1	0.334	0.112	0.126	42.2	42.2	0.752	30	0.090
	Can2	0.281	0.079	0.086	28.7	70.9	0.846	20	0.247
Blood parameters	Can1	0.755	0.570	1.325	28.0	28.0	0.040	140	<0.001
	Can2	0.747	0.557	1.260	26.7	54.7	0.092	108	0.007

d.f. = degrees of freedom.

## Data Availability

The original contributions presented in the study can be found in the article and Supplementary Material and further enquiries can be directed to the corresponding authors.

## Supplementary Materials

The following supporting information can be downloaded at: https://www.mdpi.com/article/10.3390/vetsci12060582/s1; Table S1: Descriptive statistics for the ejaculates and spermatozoa from artificial insemination (AI) doses (n = 171) obtained from fertility-proven boars (n = 6); Table S2: Descriptive statistics for the pregnant sows and their litters (n = 152) obtained after artificial insemination (AI); Table S3: Descriptive statistics for the blood biochemistry from piglets (n = 81) obtained after the artificial insemination (AI) of sows; Table S4: Descriptive statistics for the hematology from piglets (n = 81) obtained after the artificial insemination (AI) of sows; Table S5: Pearson correlations between sperm parameters; Table S6: Pearson correlations between litter parameters; Table S7: Pearson correlations between blood parameters; Table S8: Pearson correlations between sperm and litter parameters; Table S9: Pearson correlations between sperm and blood parameters; Table S10: Pearson correlations between litter and blood parameters; Table S11: Standardized canonical coefficients for the canonical variates resulting from the Canonical Correlation Analysis (CCA) using the variable sets of sperm and litter parameters. Only canonical variates from the first (significant) canonical correlation are shown (X_1_ and Y_1_); Table S12: Cross loading of the original variables with the opposite canonical variates resulting from the Canonical Correlation Analysis (CCA) using the variable sets of sperm and litter parameters. Only canonical variates from the first (significant) canonical correlation are shown (X_1_ and Y_1_); Table S13: Standardized canonical coefficients for the Canonical Discriminant Analysis (CDA) on the boar effects on sperm parameters. The first two canonical correlation are shown; Table S14: Canonical structure coefficients for the Canonical Discriminant Analysis (CDA) on the boar effects on sperm parameters. The first two canonical correlation are shown; Table S15: Standardized canonical coefficients for the Canonical Discriminant Analysis (CDA) on the boar effects on litter parameters. The first two canonical correlation are shown; Table S16: Canonical structure coefficients for the Canonical Discriminant Analysis (CDA) on the boar effects on litter parameters. The first two canonical correlation are shown; Table S17: Standardized canonical coefficients for the Canonical Discriminant Analysis (CDA) on the boar effects on blood parameters. The first two canonical correlation are shown; Table S18: Canonical structure coefficients for the Canonical Discriminant Analysis (CDA) on the boar effects on blood parameters. The first two canonical correlation are shown.
