# Supplementary material for: The Individual Variations in Sperm Quality of High-Fertility Boars Impact the Offspring Production and Early Physiological Functions
Source: Vet Sci. 2025 Jun 13;12(6):582. doi: 10.3390/vetsci12060582 (PMC12197536; doi:10.3390/vetsci12060582)
Supplement: Supplementary file 1 [file vetsci-12-00582-s001.zip › SupplMaterial Tables S1-S4 and S11-S18.pdf]

SUPPLEMENTARY MATERIAL

Table S1. Descriptive statistics for the ejaculates and spermatozoa from artificial insemination (AI) doses (n = 171) obtained from fertility-proven boars (n = 6).

Variable	Min.	1st quartile	Median	3rd quartile	Max.
Ejaculate Volume (mL)	60.0	115.0	155.0	260.0	570.0
Ejaculate Concentration (×10^6/mL)	48.0	123.0	188.0	231.0	433.0
AI Doses Concentration (×10^6/mL)	17.5	24.5	29.6	34.4	40.9
Total Motility (%)	67.0	88.0	92.0	94.0	98.0
Progressive Motility (%)	15.0	28.0	35.0	44.0	73.0
VCL (µm/s)	27.0	49.0	56.0	66.0	131.0
VSL (µm/s)	8.0	19.0	22.0	26.0	38.0
VAP (µm/s)	17.0	34.0	39.0	42.0	62.0
ALH (µm)	1.0	2.0	2.0	2.0	3.0
LIN (%)	19.0	30.0	39.0	47.0	69.0
STR (%)	37.0	49.5	59.0	67.0	87.0
WOB (%)	43.0	62.0	68.0	72.0	79.0
BCF (Hz)	4.0	7.0	7.0	7.0	9.0
Viability (%)	78.0	90.0	92.0	95.0	98.0
Acrosome Integrity (%)	89.0	93.0	95.0	96.0	100.0
Mitochondrial Activity (%)	70.0	90.0	92.0	94.0	97.0
DNA Fragmentation (%)	0.0	0.0	0.0	1.0	9.0

Table S2. Descriptive statistics for the pregnant sows and their litters (n = 152) obtained after artificial insemination (AI).

Variable	Min.	1st quartile	Median	3rd quartile	Max.
Parity	3.0	3.0	4.0	4.0	5.0
Gestation Length (days)	112.0	115.0	116.0	117.0	119.0
Total Born	3.0	17.0	20.0	22.0	31.0
Born Alive	0.0	15.0	19.0	21.0	26.0

Variable	Min.	1st quartile	Median	3rd quartile	Max.
Stillborn	0.0	1.0	2.0	4.0	13.0
Mummified	0.0	0.0	0.0	1.0	13.0
Males	0.0	9.0	11.0	13.0	19.0
Females	0.0	7.0	10.0	12.0	18.0
Sex Ratio (females/males)	0.2	0.4	0.5	0.5	0.8
Males Born Alive	0.0	7.0	9.0	11.0	17.0
Females Born Alive	0.0	6.0	9.0	11.0	16.0
Alive 24 h	0.0	14.0	17.0	19.0	22.0
Dead 24 h	0.0	0.0	2.0	3.0	9.0
Males Alive 24 h	0.0	6.0	8.0	10.0	15.0
Females Alive 24 h	0.0	6.0	8.0	10.0	15.0
Piglet Weight 24 h (kg)	0.7	1.2	1.3	1.5	2.3
Litter Weight 24 h (kg)	5.0	24.6	27.8	31.1	41.3

Table S3. Descriptive statistics for the blood biochemistry from piglets (n = 81) obtained after the artificial insemination (AI) of sows.

Variable	Min.	1st quartile	Median	3rd quartile	Max.
Total Protein (g/dL)	3.2	4.9	5.2	5.5	6.6
Albumin (g/dL)	2.2	2.8	2.9	3.2	3.8
Globulins (g/dL)	0.2	2.1	2.4	2.6	3.5
Creatinine (mg/dL)	0.3	0.9	1.0	1.1	1.9
Urea (mg/dL)	3.5	11.8	16.0	21.2	53.4
Glucose (mg/dL)	63.3	93.6	106.2	114.1	176.9
Cholesterol (mg/dL)	90.8	119.3	148.7	186.7	328.9
Triglycerides (mg/dL)	19.6	56.9	70.8	87.8	152.9
Amylase (IU/mL)	758.7	1441.2	1927.0	2535.0	4218.0
Lipase (IU/mL)	1.0	13.7	22.2	30.5	71.3
Creatinine Kinase (CK, IU/mL)	292.9	993.3	1715.0	3780.0	79260.0
Alkaline Phosphatase (ALP, IU/mL)	401.8	783.4	1004.0	1214.8	2770.4

Variable	Min.	1st quartile	Median	3rd quartile	Max.
γ-Glutamyl Transferase (GGT, IU/mL)	56.5	130.4	202.7	274.5	706.1
Aspartate Aminotransferase (AST, IU/mL)	17.6	79.1	112.1	182.0	1550.5
Alanine Aminotransferase (ALT, IU/mL)	6.9	29.6	43.4	64.0	243.4
Bilirubin (mg/ dL)	0.0	0.0	0.1	0.3	0.8
Calcium (mg/ dL)	10.0	10.9	11.3	11.6	14.1
Potassium (mmol/L)	5.2	8.1	9.1	10.5	13.2
Sodium (mmol/L)	97.3	129.8	135.0	138.0	157.0
Chlorine (mmol/L)	73.0	96.1	100.4	106.0	114.6

Table S4. Descriptive statistics for the hematology from piglets (n = 81) obtained after the artificial insemination (AI) of sows

Variable	Min.	1st quartile	Median	3rd quartile	Max.
Hemolysis (0-3)	0.0	0.0	1.0	2.0	3.0
Erythrocytes Concentration (×10^6 cells/μL)	4.3	5.6	6.0	6.3	6.8
Hematocrit (%)	20.5	32.5	35.3	37.3	42.0
Hemoglobin Concentration (g/dL)	6.0	10.5	11.3	11.9	13.6
Mean Corpuscular Volume (MCV, fL)	44.7	56.0	60.0	62.0	65.8
Mean Corpuscular Hemoglobin (MCH, pg)	12.5	17.6	18.8	19.9	21.9
Mean Corpuscular Hemoglobin Concentration (MCHCg/dL)	25.8	28.3	29.0	30.0	31.6
Cell Hemoglobin Concentration Mean (CHCM, g/dL)	28.0	30.9	31.8	32.5	33.6
Erythrocyte Distribution Width (RDW, %)	14.0	15.7	17.3	19.0	30.7
Cell Hemoglobin Content (CHC, pg)	12.1	16.1	17.4	18.0	20.2
Cell Hemoglobin Distribution Width (CHDW, pg)	2.6	2.9	3.1	3.4	4.4
Hemoglobin Distribution Width (HDW, g/dL)	1.9	2.2	2.4	2.5	3.3
Leukocytes Concentration (×10^3 cells/μL)	2.8	6.8	9.5	11.1	16.3
Neutrophils Concentration (×10^3 cells/μL)	0.7	1.7	2.7	4.3	9.0
Neutrophils (%)	13.2	24.3	29.8	37.6	65.4
Lymphocytes Concentration (×10^3 cells/μL)	1.7	4.1	5.0	6.2	9.4
Lymphocytes (%)	17.7	51.4	59.5	67.6	76.8

Variable	Min.	1st quartile	Median	3rd quartile	Max.
Monocytes Concentration (×10^3 cells/μL)	0.1	0.3	0.4	0.8	1.3
Monocytes (%)	1.6	3.7	5.2	7.4	12.7
Eosinophils Concentration (×10^3 cells/μL)	0.0	0.1	0.1	0.2	0.5
Eosinophils (%)	0.2	0.8	1.1	1.6	5.2
Basophils Concentration (×10^3 cells/μL)	0.0	0.1	0.1	0.1	0.5
Basophils (%)	0.4	0.8	1.0	1.4	3.3
Platelets Concentration (×10^3 cells/μL)	37.0	129.0	190.0	267.0	688.0
Plateletcrit (%)	0.0	0.2	0.2	0.3	1.4
Mean Platelet Volume (MPV, fL)	7.7	10.3	11.5	13.7	24.9
Platelet Distribution Width (PDW, %)	19.3	80.1	85.8	92.1	108.4
Mean Platelet Component (MPC, g/dL)	16.7	19.2	20.9	22.7	26.3
Platelet Component Distribution Width (PCDW, g/dL)	4.8	6.7	7.3	7.7	9.1
Mean Platelet Mass (MPM, pg)	1.2	1.3	1.4	1.6	2.0
Platelet Mass Distribution Width (PMDW, pg)	0.6	0.8	0.8	0.9	1.2
Large Platelets Concentration (×10^3 cells/μL)	0.0	16.0	23.0	33.0	342.0
Reticulocytes Concentration (×10^6 cells/μL)	0.0	0.2	0.2	0.3	0.6
Reticulocytes (%)	0.6	2.8	4.1	5.4	12.9
Reticulocyte Hemoglobin Content (CHr, pg)	10.9	14.0	15.2	16.7	18.6
Reticulocyte Mean Corpuscular Volume (MCVr, fL)	36.6	48.0	52.8	56.9	64.3

Table S11. Standardized canonical coefficients for the canonical variates resulting from the Canonical Correlation Analysis (CCA) using the variable sets of sperm and litter parameters. Only canonical variates from the first (significant) canonical correlation are shown (X_1 and Y_1).

X variate	X_1	Y variate	Y_1
Ejaculate Volume	0.147	Born Alive	-0.265
Progressive Motility	-0.347	Stillborn	-0.666
VCL	-0.53	Mummified	-0.262
Viability	0.114	Dead 24 h	0.815
Mitochondrial Activity	-1.22	Sex Ratio	-0.038
DNA Fragmentation	-0.538	Piglet Weight 24 h	0.033

Table S12. Cross loading of the original variables with the opposite canonical variates resulting from the Canonical Correlation Analysis (CCA) using the variable sets of sperm and litter parameters. Only canonical variates from the first (significant) canonical correlation are shown (X_1 and Y_1).

X variate	Y_1	Y variate	X_1
Ejaculate Volume	0.11	Born Alive	0.06
Progressive Motility	0.34	Stillborn	-0.27
VCL	-0.35	Mummified	-0.1
Viability	-0.23	Dead 24 h	0.25
Mitochondrial Activity	-0.28	Sex Ratio	0.01
DNA Fragmentation	0.01	Piglet Weight 24 h	0.03

Table S13. Standardized canonical coefficients for the Canonical Discriminant Analysis (CDA) on the boar effects on sperm parameters. The first two canonical correlation are shown.

Variate	Ejaculate Volume	Progressive Motility	VCL	Viability	Mitochondrial Activity	DNA Fragmentation
Can1	0.128	-0.507	0.827	0.251	0.136	-0.261
Can2	-0.912	-0.258	-0.326	0.196	-0.066	-0.333

Table S14. Canonical structure coefficients for the Canonical Discriminant Analysis (CDA) on the boar effects on sperm parameters. The first two canonical correlation are shown.

Variate	Ejaculate Volume	Progressive Motility	VCL	Viability	Mitochondrial Activity	DNA Fragmentation
Can1	-0.074	-0.597	0.808	0.177	0.210	-0.102
Can2	-0.854	-0.271	-0.186	0.220	0.186	-0.212

Table S15. Standardized canonical coefficients for the Canonical Discriminant Analysis (CDA) on the boar effects on litter parameters. The first two canonical correlation are shown.

Variate	Born Alive	Stillborn	Mummified	Dead 24 h	Sex Ratio	Piglet Weight 24 h
Can1	-0.488	-0.230	-0.104	0.089	0.200	-1.123
Can2	-0.256	-0.689	-0.24	0.863	-0.157	0.055

Table S16. Canonical structure coefficients for the Canonical Discriminant Analysis (CDA) on the boar effects on litter parameters. The first two canonical correlation are shown.

Variate	Born Alive	Stillborn	Mummified	Dead 24 h	Sex Ratio	Piglet Weight 24 h
Can1	-0.012	0.207	0.218	0.311	0.180	-0.899
Can2	0.226	-0.683	-0.337	0.582	-0.065	0.106

Table S17. Standardized canonical coefficients for the Canonical Discriminant Analysis (CDA) on the boar effects on blood parameters. The first two canonical correlation are shown.

Variate	Hemolysis	Albumin	Globulins	Creatinine	Urea	Glucose	Cholesterol
Can1	-0.375	-0.494	-0.141	-0.056	0.182	0.569	0.024
Can2	0.607	0.018	-0.030	-0.006	0.280	0.303	0.098
**Variate**	**Triglycerides**	**Amylase**	**Lipase**	**Creatinine Kinase (CK)**	**Alkaline Phosphatase (ALP)**	**γ-Glutamyl Transferase (GGT)**	**Bilirubin**
Can1	-0.108	-0.993	-0.228	0.270	0.120	0.006	-0.087
Can2	-0.308	-0.118	0.496	-0.018	0.152	-0.374	0.405
**Variate**	**Calcium**	**Potassium**	**Sodium**	**Hemoglobin**	**Erythrocyte Distribution Width (RDW)**	**Neutrophils Concentration**	**Lymphocytes Concentration**
Can1	0.441	0.179	0.208	0.481	0.606	0.946	0.566
Can2	-0.703	-0.089	0.238	0.451	0.873	0.966	1.081

Variate	Monocytes Concentration	Eosinophils Concentration	Basophils Concentration	Platelets Concentration	Platelet Distribution Width (PDW)	Mean Platelet Component (MPC)	Reticulocytes Concentration
Can1	-0.450	0.146	0.296	-0.167	0.091	-0.162	0.476
Can2	0.243	-0.002	0.094	0.110	0.084	0.354	-0.020

Table S18. Canonical structure coefficients for the Canonical Discriminant Analysis (CDA) on the boar effects on blood parameters. The first two canonical correlation are shown.

Variate	Hemolysis	Albumin	Globulins	Creatinine	Urea	Glucose	Cholesterol
Can1	-0.142	-0.007	-0.216	0.178	-0.191	0.447	-0.029
Can2	0.316	0.034	-0.105	-0.099	0.322	0.236	-0.097

Variate	Triglycerides	Amylase	Lipase	Creatinine Kinase (CK)	Alkaline Phosphatase (ALP)	γ-Glutamyl Transferase (GGT)	Bilirubin
Can1	-0.404	-0.550	-0.142	-0.096	-0.109	0.020	-0.125
Can2	-0.157	-0.184	0.354	0.185	-0.020	-0.143	0.157

Variate	Calcium	Potassium	Sodium	Hemoglobin	Erythrocyte Distribution Width (RDW)	Neutrophils Concentration	Lymphocytes Concentration
Can1	0.216	0.198	0.256	-0.060	0.129	0.033	0.018
Can2	-0.516	-0.011	-0.164	-0.263	0.368	-0.058	0.125

Variate	Monocytes Concentration	Eosinophils Concentration	Basophils Concentration	Platelets Concentration	Platelet Distribution Width (PDW)	Mean Platelet Component (MPC)	Reticulocytes Concentration
Can1	-0.333	-0.152	0.164	-0.021	0.161	-0.027	0.036
Can2	-0.127	-0.187	0.029	-0.115	0.133	0.225	-0.212
